# Continuous *in vitro* propagation of *Babesia microti*

**DOI:** 10.1128/iai.00481-23

**Published:** 2024-06-05

**Authors:** Lee Fuller

**Affiliations:** 1 Fuller Laboratories, Fullerton, California, USA; University of Pennsylvania, Philadelphia, Pennsylvania, USA

**Keywords:** *Babesia microti*, IgM, C3b complement, BmSA1

## Abstract

The currently accepted initiation of Babesia infection describes a sporozoite stage infused into the host, along with other saliva components, by the tick vector. This sporozoite can enter and initiate erythrocyte infection directly. In the particular case of *Babesia microti*, however, that sporozoite loses the ability to further propagate *in vitro* once deprived of its natural host. True *B. sensu stricto* do not require the host collaboration described in this study. Hence it has become a current topic of research involving *B. microti* (*B. sensu lato*), a rather unique species that requires host collaboration to maintain an erythrocyte propagation cycle. The main attachment protein is synthesized by this parasite in excess and exported to the host from the erythrocyte infrastructure to immunize the host at all stages of infection. The synthesis of host immune IgM antibody is necessary for the propagation of *B. microti*, being central to entry into uninfected host erythrocytes. Sequential use of the host immune system then involves complement factor C3b to complete the three-part assembly necessary to initiate the rhoptry sequence for invasion of uninfected erythrocytes and further propagation. These several components must be furnished within the *in vitro* culture medium and the sequence of these reactions is discussed. The corollary view of the parasite survival versus the host immune defenses is also discussed as it involves the same host factors promoting continuing parasite growth. This is the first description of continuous *in vitro* propagation of *B. microti*.

## INTRODUCTION

*Babesia microti* is a tick-borne Apicomplexan parasite that has been detected worldwide. Although the initial case in Massachusetts was published in 1970 (1), it remains an emerging threat, especially due to the blood-borne nature of this pathogen and its ability to remain dormant in a donor blood unit or donor organ months after being collected from an otherwise healthy donor. Although North America and China (2) currently report the most tick-borne cases (2, 3), it is also found in Europe (4, 5), South America (6), Asia and Southeastern Asia (7–9), and other parts of the world. It should be noted that these reports do not include the high levels of population immunity in endemic regions nearer to the equator where cases are rarely reported, although several field studies describe widespread sub-clinical infection, high population seroprevalence, and low incidence of clinical disease (6–9).

*Babesia microti sensu lato* forms a distinct clade within the Apicomplexa, as was apparent before publication of the full genome sequence (10) demonstrating a significantly smaller and more prototypic genome than any of the *B. sensu stricto* species. Although unable to simply attach to an uninfected host erythrocyte and enter using an evolved sequence within its apical complex, as *B. sensu stricto* do, *B. microti* has evolved a unique collaboration with its host to facilitate multiplication *in vivo*.

**Fig 1:**
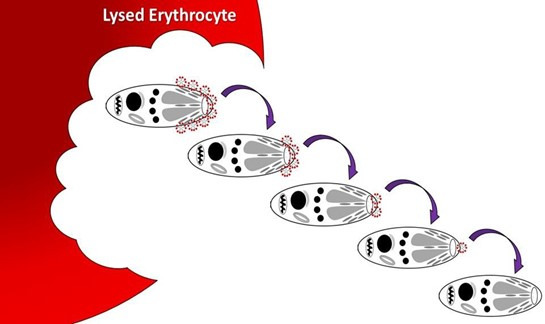
Trophozoite coating (Immune IgM and C3b) after each propagation stage following asexual replication, en route between lysed erythrocyte and invasion of an uninfected erythrocyte *in vitro. In vivo* this replacement on the trophozoite membrane is a host function, but *in vitro* this replacement on the trophozoite membrane must be included in the culture medium formulation. The pattern of 3–4 days growth, followed by rapid crash of the culture (see Fig. 2), has been repeated in all previous culture systems that do not make these host components available in the culture medium and continue to be utilized as a short-term culture method for rapid results (12, 13).

Short-term propagation of *B. microti in vitro* has been utilized successfully wherein parasites are initially generated by infecting laboratory animals. These parasites maintain a membrane coating of both immune IgM antibody and C3b complement from their propagation *in vivo*. This short-term assay should not be confused with a continuous culture, as it is understood that such cultures propagate for three cycles and then are permanently inactive. The coating of the *B. microti* outer membrane can be seen by IFA and DFA (direct antibody staining) of infected erythrocytes from hamsters and other laboratory animals. This *in vivo* coating is produced by the host defenses and allows several rounds of cell culture propagation, as this coating is divided between the daughter cells of asexual division. Thus, the initial division *in vitro* leaves each daughter merozoite with 50% of the original IgM and slightly less of the original C3b (complement), which is more heat-labile (Fig. 1). When the *in vitro* system does not replenish this coat following the release of the trophozoite to seek an uninfected erythrocyte, the short-term *in vitro* assay is thereby limited due to a lack of a sufficiently strong attachment between the trophozoite and the host erythrocyte membrane (11).

**Fig 2:**
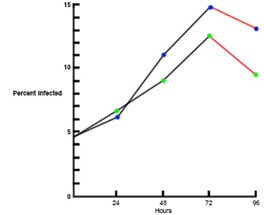
An example of timing in a short-term culture wherein *ex vivo* erythrocytes without immune IgM or fresh complement available in the culture medium. The daily sequence is 50% remaining coat at 24 h, 25% at 48 h, and 12.5% at 72 h, preceding the total inability of the parasite to further propagate (14, 15).

When I initiated this study in late 2016, much had already been learned by *in vivo* work and the short-term assays. However, two very early papers (1979) gave clues as to the basic mode of this parasite’s entry into uninfected erythrocytes. A paper in the journal Science by Yvonne Rosenberg and Charles Evans in 1979 (16) described a treatment of newborn mice to abolish all B lymphocyte production of IgM-class antibody. Compared with untreated mice the IgM-free mice were resistant to challenge with live *B. microti*. The authors concluded, “Based on the present data, the simplest explanation for the apparent lack of susceptibility to infection of suppressed mice is that … *Babesia* parasites require IgM antibody; the absence of the capacity to make IgM antibody thus confers resistance.” Yet there was no published follow-up to this rigorous study.

The possible involvement of the host complement system initially came from W.E. Chapman and P.A. Ward’s early work in 1977 (14) using sister clade *B. rhodaini*, demonstrating “a requirement for complement in the development of babesial infection in rats”. In 1981 Peter Ward and Richard Jack (15) took this a step further, involving the erythrocyte CR1 complement receptor in the attachment of *B. rhodaini*. As intriguing as these studies were, the parasite attachment to the erythrocyte CR1 membrane component was the most important to further study of *B. microti*. An attachment complex binding the *B. microti* trophozoite to the uninfected erythrocyte requires both immune IgM and the complement system, basically to assist the parasite’s attachment in an additive way with sufficient force to allow this parasite to perform the entry using its primitive apical complex. The complement protein C3, which is found in normal host serum, is fixed by the immune IgM bound to the parasite attachment protein (BmSA1) and is cleaved by the host C3 convertase enzyme to produce a bound C3b, releasing anaphylatoxin C3a. This allows initiation of the erythrocyte entry sequence (apical complex) that *B. microti* had already evolved, but the initial binding of the predominant attachment protein (BmSA1) cannot successfully initiate this sequence by itself as seen in more evolved *B. sensu stricto* species (9).

Starting with the notion that this pathogen has hijacked the host immune system successfully, I constructed this interplay *in vitro* and measured the growth of this parasite under various experimental conditions. Here, I report the use of culture media containing those factors necessary for the construction of an erythrocyte attachment complex, enabling successful propagation of *B. microti in vitro* for as long as daily media changes are maintained. If fresh medium with added attachment factors (active complement and IgM anti-BmSA1) is not available daily, the culture will show a loss of 50% propagation in comparison to daily changes. The hypothesis is that the acute phase antibody response and the interplay with the host complement components combine to build an erythrocyte attachment complex. This was also based on having visualized these components attached to the parasites grown *in vivo* (mice and hamsters) using fluorescence-conjugated anti-IgM and anti-complement C3 reagents. When these media components are not available *in vitro* following asexual reproduction of the merozoites and lysis of the host erythrocyte, these trophozoites are not replenished by the host bloodstream and can only enter an uninfected erythrocyte using the left-over portion of their membrane coat (Fig. 1). As the coating of the merozoite outer membrane is divided between the progeny of asexual replication, the *in vitro* propagation described here replaces the host function of recoating these trophozoites with IgM and C3b.

The majority of *B. microti* infections are initiated by the bite of an infected *Ixodes* tick and the injection of sporozoites in the tick saliva into a new host. These *B. microti* sporozoites have developed from gametes in the tick and are the activated form that must initiate the host immune response once introduced into the host. Although little is known about the sporozoite stage, it has been inferred that this stage initiates the host immune response without which it cannot further propagate. This topic is being investigated further because it has been hypothesized that sporozoites directly bind and enter host erythrocytes. Currently, there is a lack of experimental evidence describing an accessory tick-derived erythrocyte-binding factor. The sporozoite initially divides to form merozoites, and then they begin to synthesize and export free BmSA1, possibly via vesicles and tubules (17). When this merozoite construction is active within the host erythrocytes, it can be visualized using IFA staining specific for the BmSA1 protein. This construction occurs in a minority of infected cells, but such forms immediately begin active protein secretion.

The host immune surveillance system primarily comprised erythrocytes, monocytes, and macrophages. When parasite antigens are detected by receptors, either free or bound to trophozoites, the production of immune IgM is initiated by the host. This surveillance system utilizes the CR1 erythrocyte complement receptor to gather immune complexes of antigen (BmSA1) and complement (C3b). Although the erythrocytes bring these antigens to the regional lymph nodes, the host monocytes and macrophages also detect and remove these collected complexes from the erythrocytes and distribute this material, beginning with the local lymph nodes where the initial immune anti-BmSA1 IgM is synthesized. Macrophages also engulf parasites and immune complexes directly, distributing epitopes to regional lymphoid centers. This host IgM antibody is then utilized by the parasite to form the immune-mediated attachment complex that allows parasite propagation. The methods described below are a summary of our early work with this organism (18) with later modifications.

## MATERIALS AND METHODS

### *B. microti* isolates

Various *B. microti* isolates, including Gray, Westport, RMNS, and Naushon strains, were kindly shared by Dr. Sam Telford (Tufts University, North Grafton, MA, USA) who maintained them by alternate tick-passage and mammalian (Syrian Hamster and/or SCID mouse) passage. Although all the above strains propagated well in the *in vitro* cultures, the remainder of this work was limited to working with the Gray strain. The mouse spleen cells used to initiate the monoclonal work (see below) were also infected with this same strain. The major surface protein in these propagation studies was originally called Surface Antigen 1 and will be referred to as BmSA1.

#### Human erythrocytes

Human type “O” blood was either purchased (Innovative Research, Novi, MI, USA) or donated at 2 weeks intervals by the author and drawn by a professional phlebotomist. Anticoagulated blood (K2 EDTA) is washed 4–5 times with sterile phosphate-buffered saline (PBS), then twice with Puck’s Saline Glucose (Fisher Scientific, Los Angeles, CA, USA) with additional (10%) glucose (PSG + G) and 0.1 mg/mL gentamicin sulfate (Gibco, Grand Island, NY, USA). This erythrocyte suspension is set to a 50% cell suspension in PSG + G and stored at 2–8°C for up to 14 days.

#### Cryopreservation of isolates

Cryo-1 solution consists of 20% PVP-40 (polyvinylpyrrolidone) and 80% PSG + G (see under previous heading). Both cultured erythrocytes and Cryo-1 solution need to be cooled, followed by pellet-cultured cells, and an equal volume of Cryo-1 solution should be added to the pellet. This should be mixed gently, distributed into cryovials, and then will have to be placed in −72°C freezer overnight. Frozen cryovials are then placed in liquid Nitrogen storage. When needed for new cultures, the cryovial(s) are rapidly warmed and added directly to the new culture wells containing culture medium and erythrocytes.

#### Culture media formulations

The initial propagation of *B. microti* in the erythrocyte host cells occurred in a 50:50 combination of Leibovitz Medium and RPMI 1640 medium (Sigma-Aldrich, Burlington, MA, USA) containing a final concentration of 2 mg/mL AlbuMAX II (Gibco, Auckland, New Zealand), 1× GlutaMAX (Gibco, Grand Island, NY, USA), 0.1 mg/mL gentamicin sulfate, 2× HT Supplement (Gibco, Grand Island, NY, USA), 1× Insulin-Transferrin-Selenium Supplement (Gibco), and sodium pyruvate 1 mM. This basal medium was further supplemented with semi-purified human IgM (17, 19) from an acute phase *B. microti* donor plasma (commercial donor unit) and complement from snap-frozen human serum (Innovative Research, Novi, MI, USA). These supplements were optimized using titrations, which included IgM and serum complement ranges found in healthy adults. Cultures were incubated at 37°C in 24-well plates initially at 2% O2, 5% CO2, and 93% N2 (microaerophilic stationary phase), then moved to 5% CO2-in-air to reduce the number of crisis forms forming under hypoxic conditions.

Although microaerophilic conditions are often still used in cultures of *B. sensu stricto,* our *B. microti* cultures responded poorly. The combination currently used to replace microaerophilic conditions, consisting of 1 mM L-cysteine HCl and 0.02 mM bathocuproine sulfonate (both Sigma-Aldrich) in the medium, did not improve the *B. microti* cultures in a 5% CO2-in-air atmosphere and was not utilized.

Medium changes consist of 80%–90% volume exchanges daily or, minimally, every 2 days. Preserved human erythrocytes are added as necessary to the culture vessel(s) to maintain a final 5% hematocrit. Several medium formulations have been tried over time without significant improvement. Before filter sterilization of media formulations, the monoclonal IgM ascites (see below) and guinea pig or human serum complement supplements are added based on titrated optimal amounts. As rapidly as possible this complete medium is aliquoted for a typical medium change (~25 mL) and stored in tube racks at −70–80°C.

Shaker cultures in 125 mL Erlenmeyer flasks have also been utilized for multi-cell mixed cultures, maintaining even distribution with 100 rpm rotation within a 5% CO2-in-air incubator. Although monocytic cell lines THP-1 (human) and P388D1 (mouse) both secrete active complement and have been useful, the hybridomas producing the monoclonal IgM anti-BmSA1 tend to become non-secreting (data not shown) over time. Further trials in both shaker flasks and 24-well plates are underway using live P388D1 with daily additions of IgM monoclonal anti-BmSA1.

#### IFA testing

To monitor culture growth, microscope slides of culture material were prepared using Teflon-masked 12-well silane-treated microscope slides. Cell suspensions of 100 µL are generated by removing 1 μL of the settled erythrocyte layer and mixing it with 100 μL PBS with 1% goat serum. Suspensions are added to the 4 mm slide wells to produce a thin layer with cells almost touching. Slides are then acetone-fixed for 10 min before use.

After the slides are processed, the positive and negative human control sera are applied to these wells (9–10 µL/well) and incubated at 37°C 30 min. When the incubations are complete, the slides are washed briefly with PBS, followed by the addition of goat anti-human IgG-Alexafluor 488 conjugate (our product GPC-100). After another 30 min incubation at 37°C the slides are washed again and glass coverslips are mounted over the residual PBS.

A more specific assay was also obtained with the same slides utilizing the anti-BmSA1 monoclonal antibodies (see below) and staining with goat anti-mouse IgM-Alexafluor 488 conjugate (Jackson ImmunoResearch, West Grove, PA, USA). The stained slides are observed using a Motic BA410 fluorescence microscope with CoolLED pE-100 excitation light source, and a filter system for FITC (maximum excitation wavelength 490 nm, mean emission wavelength 530 nm), and 40× lens. A positive reaction is seen as sharply defined bright apple-green fluorescent intraerythrocytic inclusions and erythrocyte membrane coating. Photographs are recorded with the Gryphax Prokyon camera and software (Jenoptic AG, Germany).

**Fig 3:**
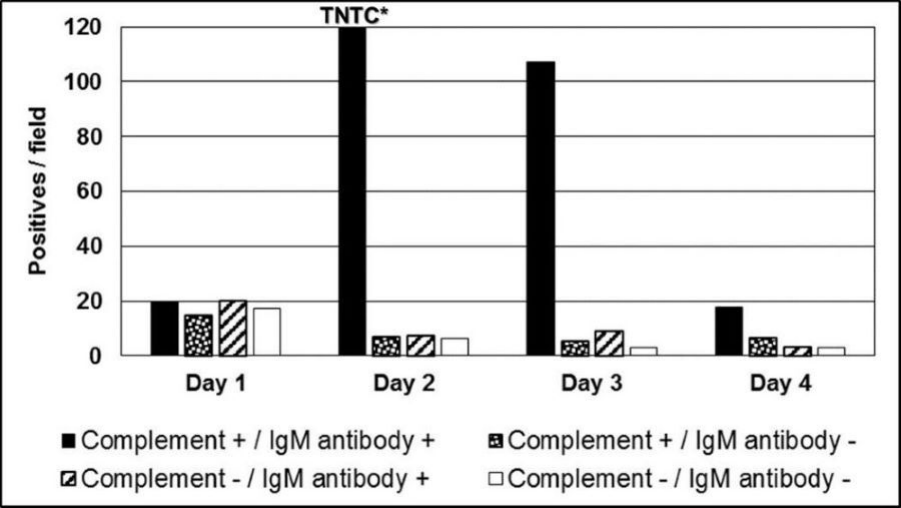
Wells of a 24-well culture plate were set on day 0 with 5–10 positive cells per 400× microscope field. On each of the 4 days, 1 µL from the settled erythrocyte layers was mixed with 100 µL PBS and counted as described for IFA testing. Components of media are listed at the bottom. Note: Result “TNTC*” refers to cell clumping (too numerous to count) (see Fig. 4). A characteristic of rapidly propagating *B. microti* is “sticky membranes” (cytoadhesion) that initially form clusters (see Fig. 6 and 7) and as noted in the above titration.

**Fig 4:**
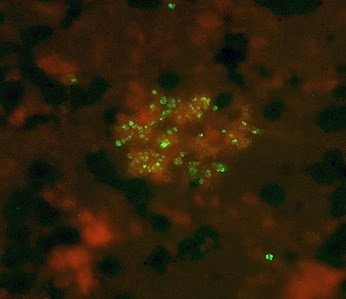
Small clusters of *B. microti* propagating in ambient oxygen (air) without THP-1 monocytes, stained with anti-*B. microti* reference serum and anti-human IgG-Alexafluor-488-conjugate.

The initial experiments were determined by titration of the optimal concentrations of human (or guinea pig) complement and IgM-class anti-BmSA1 monoclonal antibody in 24-well cell culture plates. Growth was monitored by IFA staining by fluorescence microscopy using human IgG-positive reference serum. That both complement and immune IgM were required is shown in Fig. 3. The initial attempts in 2016 cultures were maintained in this fashion continuously for 6 months before freezing them down to attend scientific meetings. At that time, unstained blood smears were sent to our domestic colleagues to perform their own Giemsa stains to confirm the morphology characteristic of *B. microti*. Resulting confirmations were an important step, and the cultures were restarted after the scientific meetings from saves stored under liquid nitrogen ( Fig. 4).

**Fig 5:**
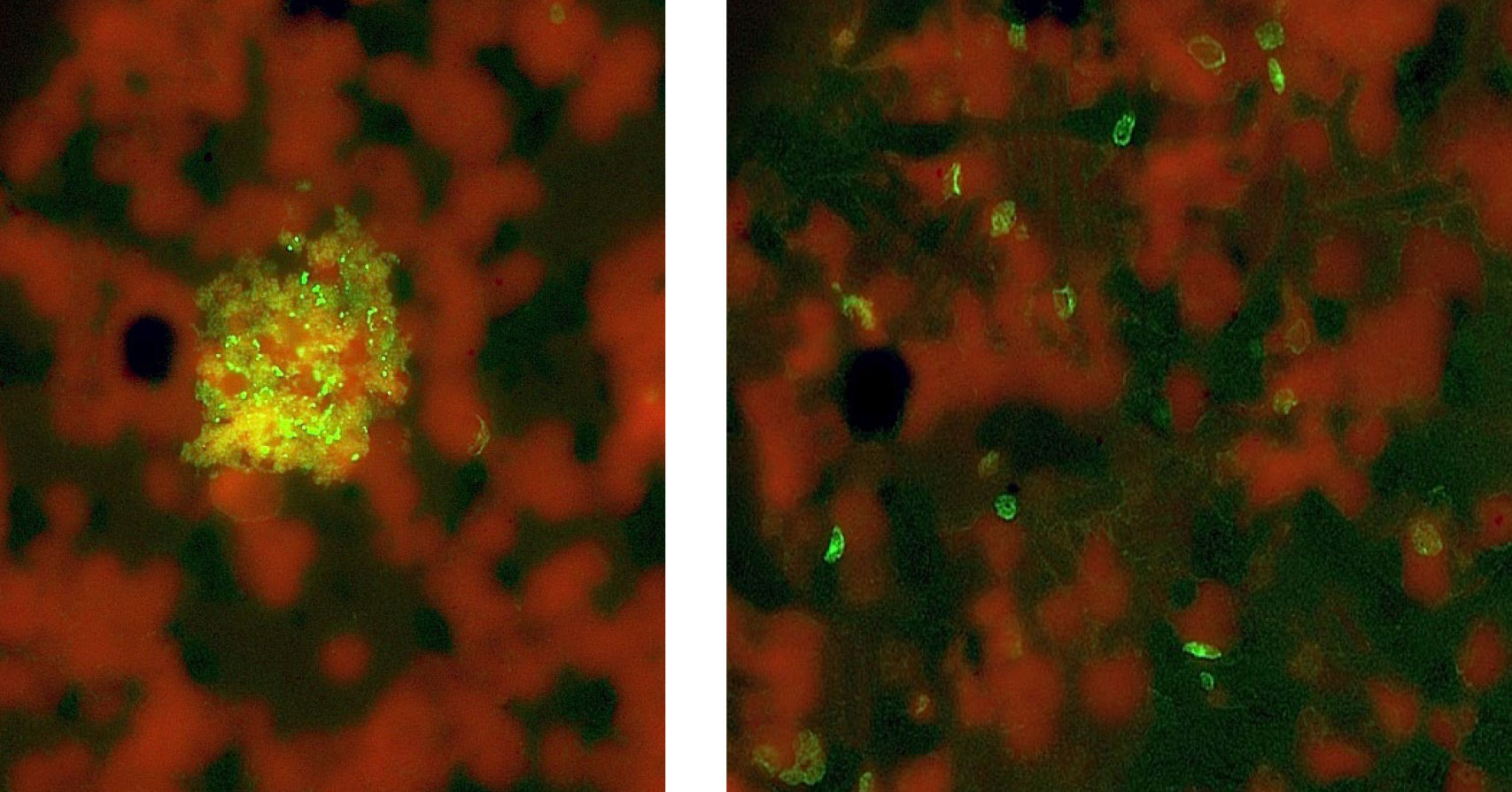
(Left) Complex clump of *B. microti* in culture with erythrocyte rim reactivity and merozoites (anti-BmSA1). (Right) Similar reactivity to anti-mouse IgM conjugate on bystander erythrocytes coated with BmSA1 protein.

### Creation of IgM-class mouse hybridomas specific for the BmSA1 protein

A series of specific hybridomas were developed against *B. microti* in collaboration with Sam Telford (Tufts University, North Grafton, MA, USA) and ImmunoPrecise Antibodies (Victoria, BC, Canada), as previously described (14). Briefly, *B. microti*-infected BALB/c splenocytes were prepared by Sam Telford and shipped to ImmunoPrecise for fusion using polyethylene glycol (PEG) with SP2/0 cells and selected for IgM-class antibody in histone acetyltransferase (HAT)-selective methylcellulose medium for 10 days.

Resulting clones were secondarily screened for reactivity to the *B. microti* merozoites by IFA in our laboratory, and then finally screened for clones that enhanced *in vitro* growth of the cultures over 2 days using media with optimal guinea pig complement. The 12 final candidate clones were back-cloned at ImmunoPrecise Antibodies for stability, and the eight surviving clones have been preserved in liquid nitrogen and/or maintained in cell culture. Monoclonal specificity was determined by western Immunoblot of *B. microti* lysate antigens and all eight clones specifically reacted with the 39 kDa protein (BmSA1).

**Fig 6:**
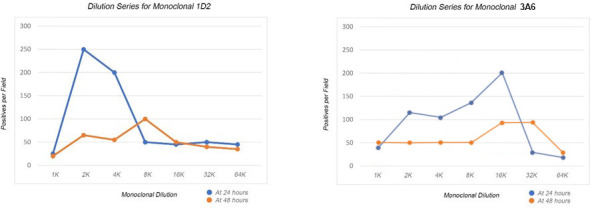
These graphs depict the peak activity of two monoclonal anti-BmSA1 antibodies at increasing dilutions at 24 h (blue) and 48 h (orange). Although the complement activity is still active, the comparison with 24 h readings defines the degradation of this activity within the second 24-h period.

Initial protocols were developed for semi-purifying the monoclonals from hybridoma culture. Because of the large amount of fetal bovine serum utilized in the hybridoma cultures, I opted for mouse ascites production at QED Biosciences (San Diego, CA, USA). Two of the eight hybridomas produced high-titer ascites antibody in significant volumes and have replaced all other sources of IgM anti-BmSA1. In accordance with IFA titers against *B. microti*, *in vitro* titration of these monoclonals began at a 1:1,000 (1k) dilution using optimal serum complement supplementation (Fig. 6). Test wells (duplicates) of the 24-well culture plates were counted by IFA staining (as before) at 24 and 48 h, without the medium change at 24 h.

### Serum complement supplement

**Fig 7:**
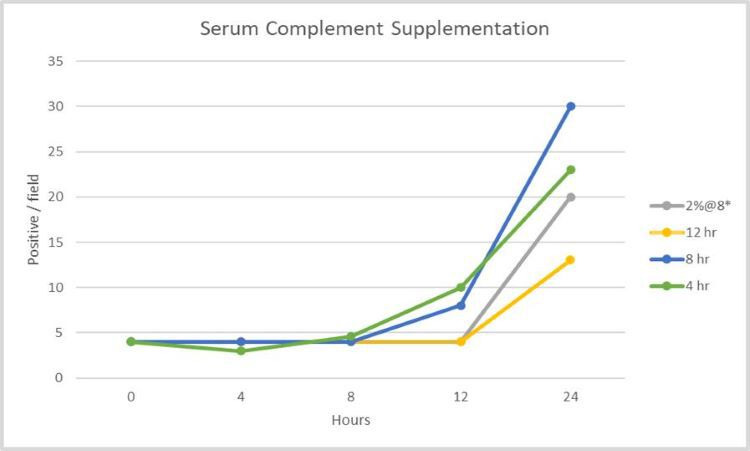
This graph shows the effect of human serum complement supplementation on *B. microti* growth. Controls without supplementation (baseline) remain at four positives per field, while another set of controls supplemented only at 4 h with 2% complement (8× compared with later supplements at 0.25%) are depicted in grey. Supplements of 0.25% complement at 4, 8, and 12 h are depicted by green at 4 h, blue at 8 h, and yellow at 12 h. Each data point is the average of triplicate wells in 24-well culture plates. Growth curves demonstrate that these infected erythrocytes can simply rest until conditions are more favorable for propagation. When serum complement factors become available, the *B. microti* return to their normal propagation cycle. These cultures had been complement-starved for 3 days before this experiment (hour 0).

To generate the attachment bridge *in vitro*, I initially added semi-purified IgM fraction (17) of an acute-phase human *B. microti* IgM serum and snap-frozen guinea pig or human serum (complement) (Fig. 7). These cultures utilized common *Babesia* medium formulations and an atmosphere of 5% CO2-in-air, combined with daily supplementation with immune IgM and serum complement. Although the IgM is still active after 2 days, the complement must be replaced daily for optimal growth. For the IgM component, I then developed eight IgM mouse hybridomas (described above) and found that they were fully capable of effectively “fixing” multiple molecules of C3 complement to the attachment complex. The final step of hybridoma screening guaranteed the specificity of each clone in demonstrating that by binding BmSA1, they were promoting parasite propagation. There were no sets of clones that demonstrated complementary activity with any other clone.

The original observations in late 2016 (13) were based on direct and indirect fluorescent antibody staining of *in vivo B. microti*-infected hamster and SCID mouse erythrocytes. Whereas infected hamster erythrocytes contained merozoites staining early in the infection by anti-hamster IgM conjugate (rabbit anti-Syrian hamster IgM-FITC [LSBio, Shirley, MA, USA]), later samples also contained IgG class antibody. In SCID mice, the intracellular antibody staining (anti-mouse-IgM antibodies) revealed only IgM class antibodies on the merozoite membrane. Additionally, the intracellular coat of the merozoites included C3 complement (anti-Syrian hamster C3, LSBio), although many fewer merozoites than those retaining the IgM coat. This observation is similar to other data showing less than 24 h stability of the serum complement proteins at 37°C.

Within the first few months of this study, I placed a co-culture containing THP-1 monocytes with *B. microti*-infected erythrocytes into an atmosphere of 2% oxygen, and the THP-1 monocytes were activated to a macrophage phenotype by this relative hypoxia. The result of this interaction, presumably cytokines, was the creation of spherical forms that ranged from dozens to thousands of pure trophozoites symmetrically arranged (spherical). I had, at that time, blocked approximately 50%–60% of the aggregate formation using monoclonals to TNF-α and interferon-β. As I did not yet have a camera with software at that time, I am finally scheduling another attempt to reproduce this dangerous form in the coming months.

## DISCUSSION

Much of the history and biology of *B. microti* and *microti*-like species (*B. vulpes*) has been hidden by a lack of a long-term *in vitro* propagation system. Distinct from the many *Babesia* parasites of human and veterinary interest (*B. sensu stricto*), *B. microti* is the most primitive species and requires something more than uninfected erythrocytes in which to propagate. Although the BmSA1 is capable of attachment, this attachment is insufficient to fully initiate the erythrocyte entry sequence (11). Previous attempts at *in vitro* propagation have been limited to short-term culture methods (12, 13). These methods have produced a 3–4-day growth phase of *ex vivo* infected blood, followed by a rapid crash due to the inability of the parasite to further invade uninfected erythrocytes. Noting that the intraerythrocytic merozoites of the *ex vivo* hamster and SCID mouse blood were coated with both IgM-class antibody and C3 complement, I attempted to re-create this *in vivo* milieu *in vitro*.

I undertook to provide a constant supplementation of BmSA1-specific IgM antibodies and a source of complement factors to an *in vitro* culture system of *B. microti* supported by human erythrocyte host cells. The presence or absence of these supplemented components had a direct correlation with the respective increase or decrease in the *in vitro* growth of *B. microti*. Taken together, the data support the hypotheses that the induction of host immune IgM response against BmSA1 and the availability of active complement proteins are both necessary for the successful transfer (entry) of daughter trophozoites into uninfected host erythrocytes.

The data supporting this sequence are found in three parts. The first part of the sequence is the parasite membrane protein BmSA1 binding an erythrocyte membrane protein. In an important paper (9), the BmSA1 is shown to bind the erythrocyte, but invasion does not take place. Immune host IgM then specifically binds the attached BmSA1 and fixes host C3 complement to this complex. The host C3 convertase enzyme is assembled and cleaves the bound C3 into C3a (anaphylatoxin) and C3b, which remains attached to the IgM. This produces a complex able to bind the trophozoite sufficiently to the erythrocyte membrane to allow the trophozoite apical complex to fulfill its invasion scenario. More advanced *Babesia* species (*sensu stricto*) do not require these sequential steps, and the evolved apical complex allows erythrocyte entry much more directly and without additional factors. An evolutionarily recent offshoot of the *B. microti* line, the Red Fox isolate (*B. vulpes*), reacts similarly to *B. microti in vitro* with our current medium and propagates in canine erythrocytes (manuscript in progress).

**Fig 8:**
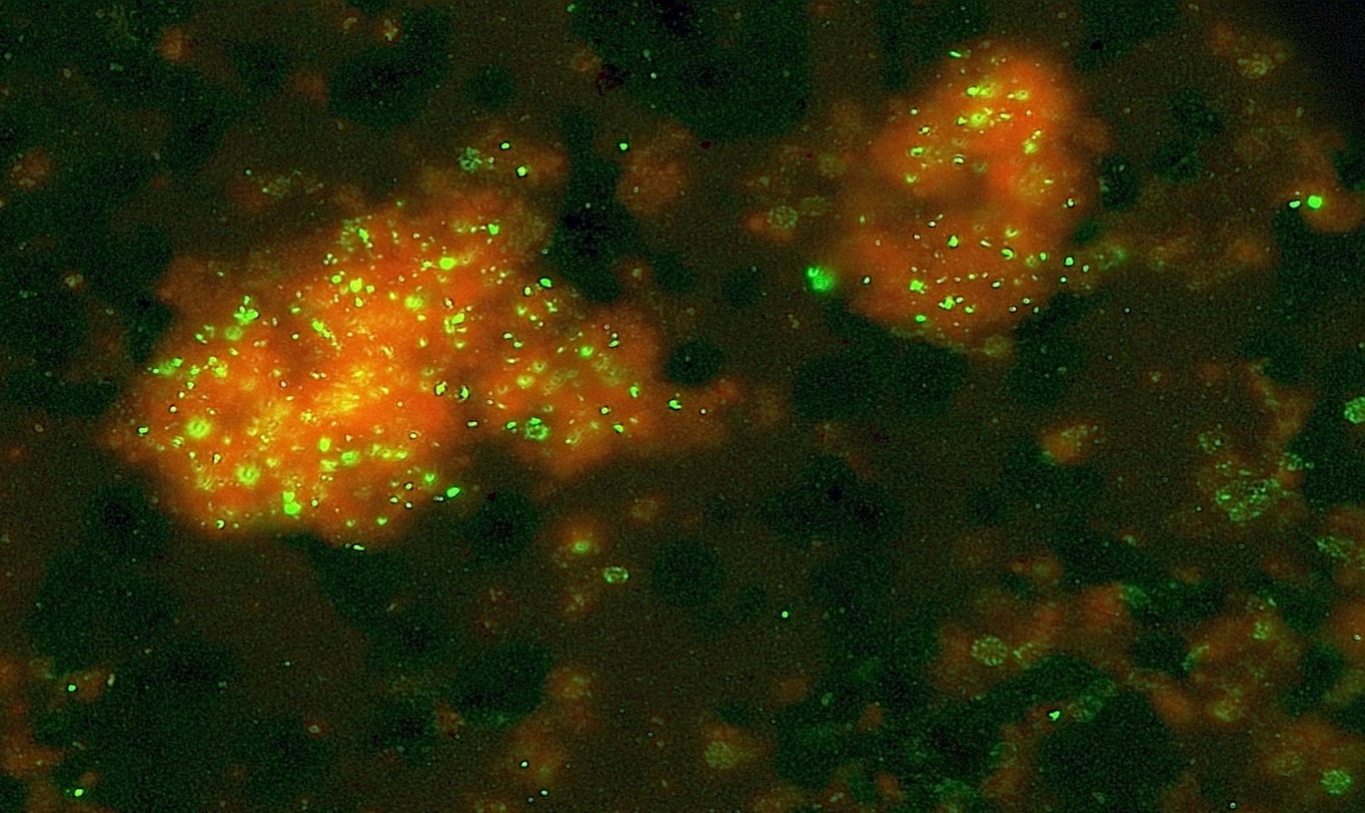
Clusters of infected erythrocytes with rim reactivity on uninfected bystander cells (anti-BmSA1 and C3b).

The alternate scenario for this sequence is the merozoite synthesis and export of free BmSA1 (17) into the host. This free attachment protein binds erythrocytes and also monocyte and macrophage receptors with immune IgM. This free antigen complex, including BmSA1 antigen, specific IgM, and complement C3b, then acts to impede the immune surveillance system of the host by blocking coordination of that system (Fig. 5 and 8). The macrophages use erythrocytes as initial detector outposts with the mission of removing these pathogens, yet the parasite functionally delays this removal function with decoys. In most cases, the balance is tipped toward the host immune system, as the erythrocyte is the most abundant blood cell, with monocytes and macrophages also very abundant in mammals. When this scenario is seen *in vitro* with P388D1 murine monocytes, for example, there is almost a balance between the beneficial effect of macrophage complement secretion increasing *B. microti* propagation and the defensive effect of macrophages engulfing the parasite in large numbers. The host immune system generally prevails *in vivo*, yet *in vitro* the blood does not circulate due to the limiting size of culture containers and this limits the availability of uncoated erythrocytes for trophozoites. This competition is totally under the control of the researcher.

A third part of this scenario primarily affects the researcher using closed-system *in vitro* propagation techniques. The delay tactic of the parasite producing and exporting false-flag immune complexes has a place in the parasite’s survival *in vivo*, although the host clears much of this rapidly through the kidney, liver, lymph nodes, and full body blood circulation. *In vitro*, however, the confined culture volume magnifies the effect by coating the erythrocyte and monocyte receptors with ever-increasing blockage (see Fig. 7, 8). The percentage of coated erythrocytes approaches 50% within the first 1–2 days of propagation as seen by anti-BmSA1 and anti-C3 antibody conjugates, removing them from the pool of uninfected potential hosts for parasite trophozoites (see fluorescent erythrocyte rims in Fig. 5B and 8).

During this process of complement fixation by the IgM bound to BmSA1 on the erythrocyte membrane, another factor emerges. That additional factor being the anaphylatoxin C3a, cleaved by the host C3 convertase enzyme, increasing the inflammatory effect on macrophages, especially within the perivascular spaces of the liver (20) and throughout the reticuloendothelial system.

The above factors still suggest that the determining factor of continued propagation *in vivo* becomes the question of how soon the host’s immune system switches from IgM- to IgG-class antibody. Several papers (12, 13) point to the beneficial effect of acute phase serum on *B. microti* growth and the opposite effect when convalescent phase serum is used. This is also the case with the monocyte/macrophage where the cellular immunity diminishes parasite growth. The inevitable endgame in these scenarios is the lymphocyte class-switch from IgM to IgG synthesis because the ability of IgG to fix complement is 100-fold less than the IgM it replaces in the host bloodstream. The parasite has essentially bought time to increase its numbers should another tick feed on this same host. The description of this delay tactic by *B. microti*, however, has been described here in the context of healthy immune-competent hosts. In truth, the neonatal, elderly, splenectomized, or immune-compromised host can be overmatched by these tactics, leading to serious short- and long-term complications. It is the author’s hope that this description of a successful propagation of these pathogens within the *B. microti* clade entices other cell culture-adept scientists to replicate some of these techniques and study the *in vitro* characteristics of these pathogens up close.
